# Genetic testing and diagnostic strategies of fetal skeletal dysplasia: a preliminary study in Wuhan, China

**DOI:** 10.1186/s13023-023-02955-4

**Published:** 2023-10-25

**Authors:** Wanlu Liu, Jing Cao, Xinwei Shi, Yuqi Li, Fuyuan Qiao, Yuanyuan Wu

**Affiliations:** Department of Obstetrics and Gynecology, Tongji Hospital, Tongji Medical College, Huazhong University of Science and Technology, No.1095 Jiefang Avenue, Wuhan, 430030 China

**Keywords:** Fetal skeletal dysplasia, Genetic testing, Prenatal diagnosis, Ultrasound scanning

## Abstract

**Background:**

Fetal skeletal dysplasia is a diverse group of degenerative diseases of bone and cartilage disorders that can lead to movement disorder and even death. This study aims to evaluate the diagnostic yield of sonographic examination and genetic testing for fetal skeletal dysplasia.

**Methods:**

From September 2015 to April 2021, the study investigated 24 cases with suspected short-limb fetuses, which were obtained from Tongji Hospital affiliated to Tongji Medical College of Huazhong University of Science and Technology. To identify the causative gene, multiple approaches (including karyotype analysis, copy number variations and whole exome sequencing) were performed on these fetuses. And further segregation analysis of the candidate variant was performed in parents by using Sanger sequencing.

**Results:**

**①** Out of 24 cases, likely pathogenic variants in *FGFR3, FBN2, COL1A2, CUL7* and *DYNC2H1* were detected in 6 cases; pathogenic variants in *FGFR3, IMPAD1* and *GORAB* were identified in other 6 cases; and variants in *WNT1, FBN1, OBSL1, COL1A1, DYNC2H1* and *NEK1*, known as Variant of Undetermined Significance, were found in 4 cases. There were no variants detected in the rest 8 cases by the whole exome sequencing. ② Of 24 cases, 12 (50%) were found to carry variants (pathogenic or likely pathogenic) in seven genes with 12 variants. Four fetuses (16.7%) had variants of uncertain significance.

**Conclusion:**

Genetic testing combining with ultrasound scanning enhances the accurate diagnosis of fatal skeletal dysplasia in utero, and then provides appropriate genetic counseling.

## Introduction

Fetal skeletal dysplasia (FSD) is one of the most common fetal malformations, of which the incidence rate is approximately 2.4–4.5 out of 10,000 births [1, 2]. It is defined as a group of bone and cartilage disorders with diverse clinical and genetic heterogeneity that can lead to movement disorder and even death. The pathogenicity is closely associated with mutations of the genes encoding collagen (e. g. type I, II, IX, IX, XI collagen) [3]. Current scientific study reported many pathogenic gene variations in the proband with skeletal dysplasia and variants occurred in causative genes of *COL1A1, COL1A2, WNT1, OBSL1, FGFR3, IMPAD1, FBN2,* and *GORAB* [4–6]. According to the report of "Nosology and Classification of Genetic skeletal disorders: 2019 Revision", 461 disorders classified within 42 different groups have been described based on radiologic, molecular and biochemical criteria [7]. The clinical phenotype is broad and ranges from a mild to a severe form. Genotype and phenotype relationship is unclear. And then an accurate diagnosis of FSD can be challenging due to its rarity and variety of phenotype, especially for fetuses.Therefore, the determination of FSD is challenging due to the diversity of FSD phenotypes, particularly in prenatal diagnosis.

So far, ultrasound is the most common imaging test to detect fetal skeletal abnormality in early pregnancy [8]. However, overlapping features and phenotypic variability of skeletal dyplasias are limited to differentiate FSD and provide a definitive diagnosis via image findings. In addition, phenotypic characteristics of FSD do not manifest until later in pregnancy, which undoubtedly increase the difficulty in definite diagnosis. Recently, with the advance in next-generation sequencing technology, high-throughput sequencing has been considered as an effective method for genetic diagnosis. E.g. whole exome sequencing (WES), is advantageous in identification of De novo and compound heterozygous variants [9, 10]. Suggested by the American College of Medical Genetics and Genomics (ACMG), next-generation sequencing can be considered to increase the sensitivity of diagnosis when the traditional gene testing, such as chromosomal microarray analysis, failed to yield a definitive result for the diagnosis [11].

By analyzing the imaging in terms of ultrasounds and gene variation of 24 cases with suspected fetal skeletal dysplasia, this study successfully obtained some solid evidence for prenatal diagnosis of FSD. Ultrasound scanning combining with genetic test in the first or second trimester of gestation enhances the accurate diagnosis of fatal skeletal dysplasia in utero, then provides appropriate genetic counseling.

## Materials and methods

### Editorial policies and ethical considerations

Our study was approved by the Research Ethics Committee of Tongji Hospital affiliated to Tongji Medical College of Huazhong University of Science and Technology (IRB reference number: M2019036). Informed consents were obtained from all the participants in the study.

### Patients information

After approval of the Research Ethics Committee of our Hospital, informed consents and samples were obtained from all the participants. Twenty-four cases were included in the study since all fetuses were detected with suspected short-limb from primary ultrasound scanning in the prenatal diagnosis center (one of prenatal diagnosis referral centers in China) of Tongji Hospital affiliated to Tongji Medical College of Huazhong University of Science and Technology between September 2015 and April 2021. Out of 24 cases, the pregnant women in case 9 and 10 had osteogenesis imperfecta, while the clinical phenotypes of the pregnant women and their spouses of the rest cases (22 cases) were all normal. In addition, case 6 had induced labor previously due to the suspected short limbs of the fetus determined only by ultrasound examination.

### Ultrasound scanning

The biometric data of the fetal skeleton (including biparietal diameter (BPD), head circumference (HC), measurements of all long bones (including humerus length (HL), femur length (FL)), assessment of skeletal mineralization), and any other abnormalities were collected and analyzed in comparison with normal values [12].

### Sample collection

Amniotic fluid and blood samples were retrieved from umbilical cord by amniocentesis and cordocentesis individually under ultrasound at second trimester of gestation. For the cases of which the blood or amniotic fluid samples could not be collected, two pieces of fetal muscle tissue (2 × 2 cm, containing the skin) or umbilical cord (about 3 cm) were sampled after the termination of pregnancy.

### Fetal karyotype analysis and genetic analysis

Karyotype analysis was performed on cultured amniotic fluid cells. Fetal genomic DNA was extracted from amniotic fluid samples or the tissues samples of fetuses using a DNA Extraction Kit (TianGen, Beijing, China) according to the manufacturer's instructions and stored at − 20 °C for further analysis. The genomic DNA of the couples in all cases was extracted from whole blood sample using the same protocol/Kit. The WES was performed for all the DNA samples. After fetal DNA was quantified with Nanodrop 2000 (Thermal Fisher Scientific, DE), 3–5 μg DNA was used for sequencing and copy number variations (CNV) test. The sequencing data was used for analyzing likely pathogenic gene variation by the Polyphen 2.0 and SIFT software [13, 14]. Chromosome profiles were finally plotted as copy number (Y-axis) vs. 20- kb count windows (X-axis).

Identified and mapped CNVs were interrogated against publicly available databases, including Decipher, Database of Genomic Variants (DGV), 1000 genomes, and Online Mendelian Inheritance in Man (OMIM), and their pathogenicity assessed according to the guidelines outlined by the American College of Medical Genetics (ACMG) for interpretation of sequence variants. Variants were classifified as either pathogenic, likely pathogenic, variants of uncertain signifificance (VUS), likely benign, or benign [15].

### Familial validation

Once the pathogenic gene variations were detectable in the fetal samples, the coding regions of the mutations were identified and further examined for parents by Sanger sequencing [16].

## Results

### Clinical information and ultrasound findings

Ultrasound imaging revealed normal amniotic fluid volumes and normal appearance of the brain, heart, liver, or kidneys in all cases, whereas the suspected skeletal malformations were detected. The lengths of humerus and femur in 24 cases were found to be either significantly lower than the reference (Mean ± SD) at the same gestation period according to Hadlock’s reference chart. The results of prenatal ultrasound were summarized in Table 1.

**Table 1:** Ultrasound findings in this study

No.	Age	Gestation/parity (G/P)	Gestation (weeks)	HL (cm)		FL (cm)		BPD (cm)	HC (cm)	Skeletal malformation
				Value M ± SD		Value M ± SD				
1	39	G1P0	30^+2^	4.3	5.2-4SD	4.5	5.8-5SD	7.7	27.4	Unseen
2	24	G1P0	17^+5^	1.0	2.4-8SD	1.1	2.4-5SD	3.9	14.7	Unseen
3	21	G1P0	32	4.6	5.5-5SD	4.9	6.2-5SD	8.3	29.3	Unseen
4	36	G1P0	33^+4^	5.0	5.5-3SD	5.6	6.2-2SD	8.2	28.2	Unseen
5	31	G1P0	23^+2^	3.2	3.9-3SD	2.9	4.1-6SD	5.5	21.3	Angle bending of ribs, left lower limb bone and humerus
6	29	G2P0	32	3.7	5.5-10SD	4.5	6.2-7SD	8.8	29.9	Unseen
7	23	G1P0	24^+5^	2.9	4.1-6SD	3.7	4.4-3SD	5.1	21	Scoliosis, bipedal varus, bipedal toes continuously hooked, bilateral humerus, tibia, fibula bent
8	29	G1P0	30	4.6	5.1-3SD	4.8	5.8-3SD	8.1	28.4	Bilateral femoral curvature
9	35	G1P0	22 + 5	3.1	3.6-3SD	3.0	3.9-3SD	5.5	19.8	Right femoral curvature
10	25	G1P0	21	3.5	3.5	3.4	3.4	5.0	18.6	Unseen
11	27	G1P0	22	2.6	3.6-5SD	2.7	3.9-5SD	5.3	19.6	Unseen
12	30	G1P0	32 + 5	4.3	5.5-6SD	4.3	6.2-7SD	8.9	28.4	Small thorax
13	32	G1P0	32	5.0	5.5-3SD	5.5	6.2-3SD	8.4	29.3	Unseen
14	29	G1P0	25	3.6	4.2-3SD	3.9	4.6-2SD	6.4	23.4	Unseen
15	29	G2P0	33	4.1	5.5-7SD	4.4	6.2-7SD	8.3	29.9	Unseen
16	30	G2P0	38	5.6	6.1-2SD	6.4	7.1-3SD	9.3	32.6	Unseen
17	30	G1P0	40 + 2	5.0	6.1-5SD	5.1	7.1-8SD	7.3	26.3	Unseen
18	33	G3P0	25 + 4	3.2	4.2-5SD	3.5	4.6-4SD	5.23	18.62	Scoliosis
19	33	G3P0	34 + 5	5.4	5.6-2SD	6.1	6.5-2SD	8.8	30.9	Unseen
20	30	G1P0	15 + 6	1.0	1.8-9SD	1.1	1.8-5SD	3.3	11.7	Small thorax, equinus
21	35	G4P1	36	5.6	5.9-2SD	6.4	6.8-2SD	9.0	32.1	Unseen
22	29	G3P1	16	1.3	2.1-5SD	1.3	2.1-4SD	3.7	13.5	Bilateral temporal bone depression
23	32	G1P0	22	2.2	3.6-7SD	2.3	3.9-6SD	5.7	21	Unseen
24	30	G1P0	30 + 5	4.7	5.1-2SD	5.0	5.8-3SD	7.7	29	Unseen

### Abnormalities by karyotype analysis and copy number variations (CNV)

The chromosome G band karyotype revealed negative in all 24 cases, while CNV was undetected in 22 cases except of case15 and 23. In the case15, we found a 0.2 MB duplication in the chromosome 7 q11.21, considered benign according to the available evidence; In the case 23, a 0.4 MB duplication in the chromosome 18p11.31q11.23 was detected and known as VUS.

### Abnormalities detected by whole exome sequencing (WES)

Out of 24 cases, 6 cases were identified with carrying likely pathogenic variants in gene *FGFR3, FBN2, COL1A2, CUL7* and *DYNC2H1*; 6 other cases were considered to carry pathogenic variants and the chromosomes of fetuses were trisomy 13 and trisomy 18, respectively; 4 cases were detected with variants in gene *WNT1, FBN1, OBSL1, COL1A1, DYNC2H1* and *NEK1*, known as VUS; The rest 8 cases showed negative in WES (Table 2), which was further confirmed by Sanger sequencing.

**Table 2:** Variants of the fetuses identified in the study

No.	Bone gene encoded	Nucleotide mutation	Amino-acid change	Heterozygosity	Mutation type	Inheritance	Inheritance type	Pregnancy Outcomes
1	*FGFR3*	c.1015C > T	p. Arg339Ter	Het	Likely pathogenic	De novo	AD	Induced labor
2	*WNT1*	c.1027G > C	p. Glu343Gln	Het	VUS	Paternal	AR	Induced labor
3	*FGFR3*	c.1144G > A	p. Gly282Arg	Het	pathogenic	De novo	AD	Induced labor
4	*FBN1* *OBSL1*	c.7842 T > A c.2135-3_2135-2delCA	p. Ala2614Ala p.2135-3_2135-2delCA	Het	VUS	Biparental	AD AR	Vaginal delivery
5	*COL1A1*	c.824G > A	p. Gly275Asp	Het	VUS	De novo	AD	Induced labor
6	*IMPAD1*	c.700G > T CDS4-5del	p.E234* CDS4-5 del	Hom Het	pathogenic	Biparental	AR	Cesarean Section(death)
7	*FBN2*	Exon21-25del	Exon21-25del	Het	Likely Pathogenic	De novo	AR	Induced labor
8	*DYNC2H1* *NEK1*	c.2641G > T c.859C > G	p. Asp881Tyr p. Pro287Ala	Het	VUS	Biparental	AR	Induced labor
9	*COL1A2*	c.1118G > C	p. Gly373Ala	Het	Likely pathogenic	Maternal	AD	Induced labor
10	*GORAB*	c.178C > T	p. Arg60*	Het	pathogenic	Maternal	AR	Cesarean section
11	*N*	N	N	N	N	N	N	Cesarean section
12	*FGFR3*	c. 1138 G > A	p. Gly380Arg	Het	pathogenic	De novo	AD	Induced labor
13	*N*	N	N	N	N	N	N	Vaginal delivery
14	*N*	N	N	N	N	N	N	Vaginal delivery
15	*CUL7*	c.3355 + 5G > A c.3722_3749dup	p. V1252Gfs*23	Het	Likely pathogenic	Biparental	AR	Induced labor
16	*N*	N	N	N	N	N	N	Vaginal delivery
17	*N*	*N*	*N*	*N*	N	N	N	Vaginal delivery
18	*DYNC2H1*	c.4072C > T	p. Arg1358Cys	Het	Likely pathogenic	De novo	AR	Induced labor
19	*N*	N	N	N	N	N	N	Cesarean section
20	*FGFR3*	c.2420G > C	p.*807Sext*101	Het	Likely pathogenic	De novo	AD	Induced labor
21	*N*	N	N	N	N	N	N	Cesarean section
22	*FGFR3*	c.1948A > G	p. Lys650Glu	Het	pathogenic	De novo	AD	Induced labor
23	*FGFR3*	c.742C > T	p. Arg248Cys	Het	pathogenic	De novo	AD	Induced labor
24	*N*	N	N	N	N	N	N	N

“N” in Table 2 means negative findings in this case

**Fig. 1:**
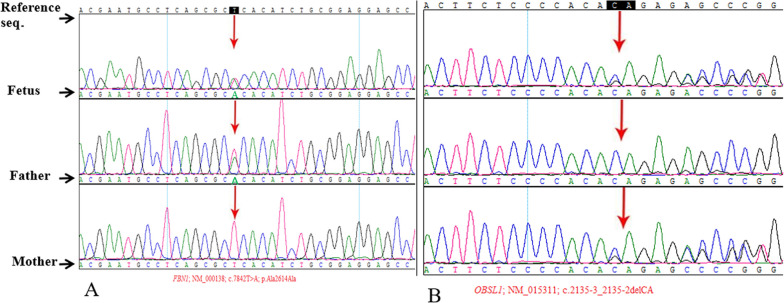
Case 4:** A** The fetus carried the c.7842 T > A (p. Ala2614Ala) mutation in the *FBN1* gene.** B** The deletion of c.2135-3_2135-2delCA in the *OBSL1* gene was detected. The Sanger verification revealed that the mutation was carried by the father while the deletion was carried by the mother

For the fetus of case 4 carried the c.7842 T > A (p. Ala2614Ala) variant in *FBN1* gene and the deletion of c.2135-3_2135-2delCA in *OBSL1* gene. The Sanger sequencing analysis further revealed that the variant was carried by the father and the deletion was carried by the mother (Fig. 1).

**Fig. 2:**
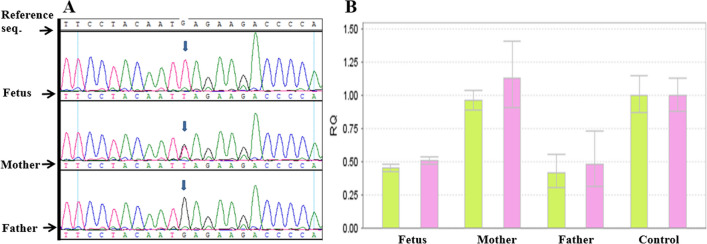
Case 6: **A** The fetus carried the c.700G > T (p. E234*) mutation in the *IMPAD1* gene and inherited from mother by Sanger Sequencing verification. **B** The deletion of CDS4-5 in *IMPAD1* gene was detected in the fetus and inherited from father by qPCR verification

For the fetus of case 6, we detected the variant of c.700G > T (p.E234*) and the deletion of CDS4-5 in *IMPAD1* gene. Both Sanger sequencing and qPCR determined that the heterozygous deletion of CDS4-5 and the homozygous variant in gene *IMPAD1* were inherited from their parents, resulting to a composite heterozygous variant (Fig. 2).

**Fig. 3:**
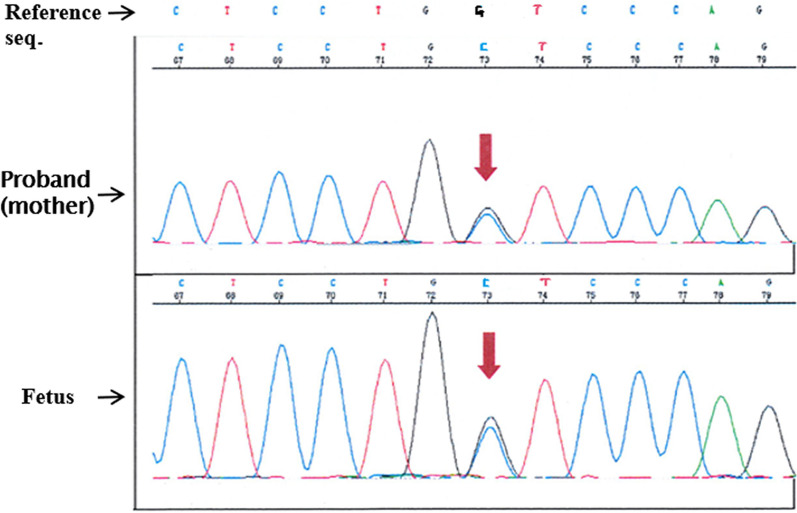
Case 9: The variant of c.1118G > C (p. Gly373Ala) in *COL1A2* gene of the fetus and the mother

**Fig. 4:**
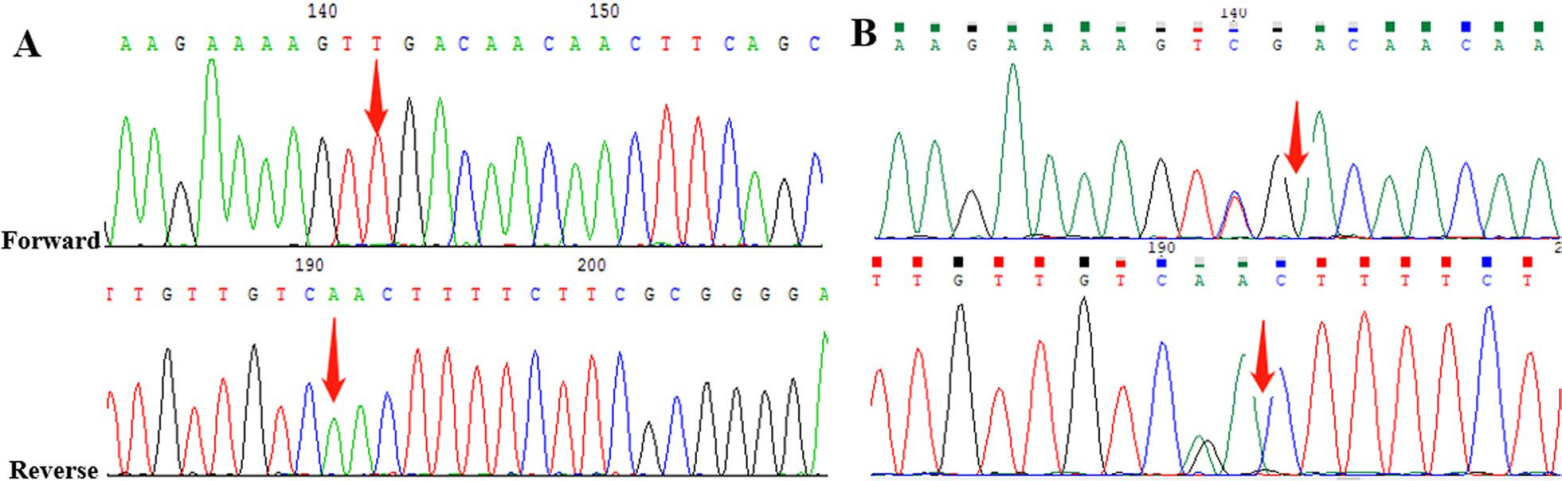
Case 10: **A** The variant of c.178C > T (p. Arg60*) in *GORAB* gene of the mother. **B** The heterozygous mutation in GORAB gene of the fetus

In the case 9 and 10, the two women were diagnosed with osteogenesis imperfecta before pregnancy. Our analysis detected the variant of c.1118G > C (p. Gly373Ala) in *COL1A2* for the fetus in case 9, and variant of c.178C > T (p. Arg60*) in *GORAB* for the fetus in case 10. The two variants originated from osteogenesis imperfecta and inherited from their mother (Fig. 3, 4).

## Discussion

The prenatal diagnosis of FSD is important at the second trimester of gestation, however, it is still challenging due to diverse clinical and genetic heterogeneity of the disorder. For decades, ultrasound is widely used in the noninvasive detection of FSD. Pajkrt and Chitty [17] found that FL and HL of the fetuses with skeletal dysplasia were 5% shorter than the normal value. Previous reports in China demonstrated that the diagnosis accuracy of continuous sequential follow-up ultrasound was over 80% and 87.2% in the second trimester of gestation [18]. In this study, by ultrasound scanning, we found the HL and FL of the fetuses in the 24 cases was either lower or higher than the normal value except of case 10 (the woman with OI (osteogenesis imperfecta) before pregnancy). The BPD and HC of the fetus were normal in all the 24 cases. In one-year follow-up survey, we found the 8 infants (account for 33.3%) were normal in skeletal development after delivery when the HL and FL of these fetuses was 2SD-3SD lower than the mean value. Differently, when the HL and FL of the fetuses was 3SD less or 3SD more, only two newborns (8.33%) were normal. The results of this ultrasound-only dependent approach are inadequate for diagnosing the disorder and impossible to differentiate the complex types of FSD.

In recent years, NGS (WES and WGS, Whole exome sequencing and Whole gene sequencing) has been applied in the area of disease diagnosis [19]. The variants detection tool needs to optimize since the detection rates of WES could be variable depending on many factors, such as the sample size, the analysis criteria, proband-only or trio WES, and so on [10]. More importantly, the complex of genetic variants was found to be associated with the diverse pathogenicity of FSD.In this study, WES identified well-described variants (pathogenic or likely pathogenic) in 12 out of 24 cases (50%), and VUS in 4 cases(16.67%), rendering a total diagnostic yield of 66.67%. Compared with our findings, three recent cohort studies reported a significantly higher diagnostic rate by WES, which was 80% (12 out of 15), 85% (11 out of 13), and 70% (21 out of 30), respectively [20–22].

In case 4, The fetus carried the c.7842 T > A (p. Ala2614Ala) variant in the *FBN1* gene and the deletion of c.2135-3_2135-2delCA in *OBSL1* gene. The mutation is synonymous mutation and the deletion is in the intron. This variant in *FBN1* gene [23] is reported to be associated with Weill Marchesani syndrome (clinical manifestations are short limb deformity, secondary glaucoma, short stature, etc.). The *OBSL1* variant [24] is related to 3-M syndrome (clinical manifestations include severe intrauterine growth retardation, short stature, recessive spina bifida, compression deformation of long metaphysis). The inheritance patterns of Weill Marchesani syndrome and 3-M syndrome are both AR (autosomal recessive inheritance). The Sanger analysis [16] indicated that the variant was carried by the father and the deletion was carried by the mother (Fig. 1). The mother of case 4 had natural delivery by vaginal at 39^+6^ weeks of gestation, and the newborn did not show any abnormality of bone development (up to 18-monthes old) at the end of the following-up survey.

In case 6, we detected the variant of c.700G > T (p.E234*) and the deletion of CDS4-5 in *IMPAD1* gene for the fetus. The heterozygous deletion of CDS4-5 and the homozygous variant in *IMPAD1* were inherited from their parents, as a compound heterozygote variant (Fig. 2). *IMPAD1*-related chondrodysplasia is an autosomal recessive disease [25]. The pregnant women had cesarean section at 38 weeks of gestation in this case, and the newborn had typical short limb deformity and died within one month after delivery.

In the study, there were two women (case 9 and 10) diagnosed with osteogenesis imperfecta before pregnancy. We detected the variant of c.1118G > C (p. Gly373Ala) in *COL1A2* for the fetus in case 9, and found the variant of c.178C > T (p. Arg60*) in *GORAB* for the fetus in case 10 (Fig. 3, 4). The two variants related to osteogenesis imperfecta and inherited from their mother, but one genetic type is autosomal dominant (AD) [26] and the other is autosomal recessive (AR) [27]. The fetus of case 9 died before delivery at 35 weeks of gestation, while the newborn of case 10 had no significant abnormality in bone development up to 2-years old in the following-up survey.

In the case 2, 5 and 8, we found the *WNT1*or *COL1A1*-related VUS in osteogenesis imperfecta, and *DYNC2H1* and *NEK1*-related asphyxiative hypoplasia of thorax. Notably, the fetuses in these cases all had short lower limbs, which was determined after abortion, while the proteins encoded by the gene variants were predicted to be deleterious using the SIFT and Polyphen analysis. Furthermore, we had negative findings by WES in 8 cases, and the 8 infants were normal in skeletal development after delivery in the telephone follow-up. Additionally, our study has some limitations. For instance, this is a single-center study with a relatively small case number and selected population. Prospective multicenter studies with large sample sizes are needed to obtain more reliable data.

## Conclusions

In sum, skeletal dysplasia is mostly hereditary. Our study has obtained 16/24 (66.67%) cases carrying 12 different skeletal dysplasia genotypes, which will broaden the spectrum of FSD in Chinese patients. Genetic diagnosis combining with ultrasound scanning enhances the accurate diagnosis of FSD in utero, then can provide appropriate genetic counseling.

## Data Availability

The datasets generated and analyzed during the current study are not publicly available due to patient privacy and confidentiality. Anonymized data can be made available from the corresponding author upon reasonable request.

## Abbreviations

FSD: Fetal skeletal dysplasia
FGFR3: Fibroblast growth factor receptor3
FBN1: Fibrillin-1
FBN2: Fibrillin-2
COL1A1: Collagen type I alpha 1 chain
COL1A2: Collagen type I alpha 2 chain
CUL7: Cullin-7
DYNC2H1: Dynein cytoplasmic 2 heavy chain 1
IMPAD1: Inositol monophosphatase domain containing 1
WNT1: Wnt family member 1
GORAB: Golgi-associated Rab-binding protein
OBSL1: Obscurin like cytoskeletal adaptor 1
NEK1: NIMA (never-in-mitosis A)-related kinase 1
VUS: Variant of undetermined significance
WES: Whole exome sequencing
WGS: Whole gene sequencing
NIPT: Non-invasive prenatal test
BPD: Biparietal diameter
HC: Head circumference
HL: Humerus length
FL: Femur length
NT: Nuchal translucency
CNV: Copy number variations
OI: Osteogenesis imperfecta
NGS: Next-generation sequencing
AR: Autosomal recessive inheritance
AD: Autosomal dominant inheritance
